# Unmasking the complexity of species identification in Australasian flying-foxes

**DOI:** 10.1371/journal.pone.0194908

**Published:** 2018-04-10

**Authors:** Linda E. Neaves, Melissa Danks, Matthew J. Lott, Siobhan Dennison, Greta J. Frankham, Andrew King, Mark D. B. Eldridge, Rebecca N. Johnson, Anja Divljan

**Affiliations:** 1 Australian Centre for Wildlife Genomics, Australian Museum Research Institute, Sydney, New South Wales, Australia; 2 Royal Botanic Garden Edinburgh, Edinburgh, United Kingdom; 3 Terrestrial Vertebrates, Australian Museum Research Institute, Sydney, New South Wales, Australia; CSIRO, AUSTRALIA

## Abstract

*Pteropus* (flying-foxes) are a speciose group of non-echolocating large bats, with five extant Australian species and 24 additional species distributed amongst the Pacific Islands. In 2015, an injured flying-fox with unusual facial markings was found in Sydney, Australia, following severe and widespread storms. Based on an initial assessment, the individual belonged to *Pteropus* but could not be readily identified to species. As a consequence, four hypotheses for its identification/origin were posited: the specimen represented (1) an undescribed Australian species; or (2) a morphological variant of a recognised Australian species; or (3) a hybrid individual; or (4) a vagrant from the nearby Southwest Pacific Islands. We used a combination of morphological and both mitochondrial- and nuclear DNA-based identification methods to assess these hypotheses. Based on the results, we propose that this morphologically unique *Pteropus* most likely represents an unusual *P*. *alecto* (black flying-fox) potentially resulting from introgression from another *Pteropus* species. Unexpectedly, this individual, and the addition of reference sequence data from newly vouchered specimens, revealed a previously unreported *P*. *alecto* mitochondrial DNA lineage. This lineage was distinct from currently available haplotypes. It also suggests long-term hybridisation commonly occurs between *P*. *alecto* and *P*. *conspicillatus* (spectacled flying-fox). This highlights the importance of extensive reference data, and the inclusion of multiple vouchered specimens for each species to encompass both intraspecific and interspecific variation to provide accurate and robust species identification. Moreover, our additional reference data further demonstrates the complexity of *Pteropus* species relationships, including hybridisation, and potential intraspecific biogeographical structure that may impact on their management and conservation.

## Introduction

*Pteropus* are a speciose group of non-echo locating bats containing 63 of the ~200 species of old world fruit bats (Pteropodidae) or flying-foxes [1]. In Australia there are five extant *Pteropus* species, including one on Christmas Island, and an additional 24 *Pteropus* species are distributed amongst the Pacific Islands. Several species are distributed across multiple island groups while others are endemic [1,2] (Table 1).

**Table 1:** Distribution of *Pteropus* species within Oceania.

	Australia	Papua New Guinea	Melanesia				Polynesia					Micronesia			
			Fiji	New Caledonia	Solomon Islands	Vanuatu	Cook Islands	Samoa	Tonga	Wallis and Futuna	Niue	Micronesia	Mariana Island	Palau	Guam
*P*. admiralitatum*		**✓**			**✓**										
*P*. alecto*	**✓**	**✓**													
*P*. anetianus*						**✓**									
*P*. capistratus*		**✓**													
*P*. *cognatus*					**✓**										
*P*. conspicillatus*	**✓**	**✓**													
*P*. *fundatus*						**✓**									
*P*. gilliardorum*		**✓**													
*P*. *howensis*					**✓**										
*P*. hypomelanus*		**✓**			**✓**										
*P*. macrotis*	^a^	**✓**													
*P*. mahaganus*		**✓**			**✓**										
*P*. mariannus*												**✓**	**✓**		**✓**
*P*. *melanotus*	**✓**														
*P*. molossinus*												**✓**			
*P*. neohibernicus*	^b^	**✓**													
*P*. nitendiensis*					**✓**										
*P*. ornatus*				**✓**											
*P*. pelewensis*														**✓**	
*P*. poliocephalus*	**✓**														
*P*. rayneri*		**✓**			**✓**										
*P*. *rennelli*					**✓**										
*P*. samoensis*			**✓**					**✓**							
*P*. scapulatus*	**✓**														
*P*. tonganus*		**✓**	**✓**	**✓**	**✓**	**✓**	**✓**	**✓**	**✓**	**✓**	**✓**				
*P*. tuberculatus*					**✓**							**✓**			
*P*. ualanus*												**✓**			
*P*. *vetulus*				**✓**											
*P*. woodfordi*					**✓**										

* indicates species with DNA sequences available

^a^ Specimens of *P*. *macrotis* from Torres Strait have subsequently been identified as *P*. *scapulatus* [2].

^b^ single occurrence on Thursday Island.

In April 2015, a flying-fox (*Pteropus* sp.), which could not readily be identified to species based on appearance, was found in Sydney following large and widespread storms. Australian *Pteropus* are well-characterised and, although morphological variation in several species can make identification of animals in the canopy difficult, it is generally straightforward with the animal/specimen in hand [3,4]. The deceased animal was donated to the Australian Museum Terrestrial Vertebrate Collection in the hope the taxonomy and potential origins of the animal could be determined. Based only on initial assessments of the external morphology of the animal, four potential hypotheses were posited. It could represent (1) an undescribed Australian species; or (2) a morphological variant of a recognised species; or (3) a hybrid individual; or (4) a vagrant from nearby Pacific Islands.

Australian *Pteropus* species occur in sympatry in many parts of their distributions [5]. Although species form mixed colonies, there can be differences in how each species partition resources such as roosting sites (e.g. different section of a colony site, and/or vertical stratification in the same trees (A. Divljan pers. comm.)) [3,5,6]. Despite this, hybridisation between species does occur, and has been recorded between *P*. *alecto* and *P*. *poliocephalus*, as well as between *P*. *alecto* and *P*. *conspicillatus* [6]. Webb and Tidemann [6] showed evidence of fertile hybrids between *P*. *alecto* and *P*. *poliocephalus*, which typically appeared morphologically similar to *P*. *alecto*, with black colouration on the head and body, but the legs were furred to the ankle—a trait typical of *P*. *poliocephalus*. These hybrids could also be distinguished genetically [6]. In *P*. *alecto* and *P*. *conspicillatus* morphologically distinguishing hybrids was inconclusive despite several key characteristics including the eye-ring of *P*. *conspicillatus*, and differences in ear shape and dentition in the two species [7]. Natural variation in morphology within species made hybrids difficult to identify confidently; even the distinctness of the eye-ring shows considerable variation within *P*. *conspicillatus* and lighter colouring around the eyes also occurs in *P*. *alecto* [8]. In contrast to hybrids of *P*. *alecto* and *P*. *poliocephalus*, genetic assessment failed to confidently identify hybrids of *P*. *alecto* and *P*. *conspicillatus* due to the degree of genetic similarities between these species at the limited number of allozymes investigated [6]. More recently, hybridisation between these two species has been hypothesised based on the distribution and relationships of mtDNA haplotypes in areas of sympatry [3].

As large bats (~600–1000 g), *Pteropus* are highly mobile and multiple instances of long distance dispersal have been reported. For example, movements of up to 500 km have been recorded over 48 h in *P*. *poliocephalus* [9]. Furthermore, several species have been observed moving between islands, including *P*. *alecto*, which has been observed moving between Australia and Papua New Guinea (PNG), between PNG and Indonesia, as well as across Torres Strait [10]. Some long distance movements also appear to be facilitated by wind [11]. At the time the unknown *Pteropus* in this study was found, two east coast lows had developed off the southeast coast of New South Wales causing widespread, prolonged and severe winds, with average speeds exceeding 50 km/h for 44 consecutive hours and gusts as high as 135 km/h in the Sydney region [12]. Thus, given this extreme storm activity there is a strong possibility that the individual represents a vagrant species from elsewhere in Australia or nearby Pacific Islands (Table 1).

To clarify the identity (and potential origin) of the atypical *Pteropus*, we undertook a detailed morphological assessment of external characteristics and genetic analysis using both mitochondrial and nuclear genes. In doing so, we significantly added to the existing vouchered reference data for these genes in several Australian and Pacific Island *Pteropus* species, which, prior to this study, were limited to only a few individuals per species. We discuss the potential drawbacks associated with species identification in complex groups based on limited sample size that were highlighted by our findings.

## Methods

### Sample location

The unknown *Pteropus* was found in Gladesville, northwest Sydney, Australia (33° 50' 08" S, 151° 08' 23" E), approximately 200 m from a known flying-fox colony, on the morning of the 25^th^ of April, 2015. The Australian Wildlife Rescue Organisation (WIRES) rescued the animal and took it to a veterinarian for assessment that day. Upon detailed examination, the veterinarian euthanized the animal under routine veterinary procedures, because of the extensive wing injuries. Subsequently, WIRES donated the flying-fox to the Australian Museum Terrestrial Vertebrate Collection on the 30^th^ of April, 2015.

### Morphological methods

The individual was sexed, and the approximate age was determined based on tooth wear, nipple condition, and general size and appearance. Body mass and standard external morphology measurements were taken, including forearm length, tibia length and ear length. The animal was photographed and examined externally for typical identification characters including pelage colour, presence of fur on the ankles and ventral wing surface, length and shape of the ears and rostrum. External body injuries and markings were also recorded. A ventral incision was made to collect liver and pectoral muscle tissue samples before the animal was fixed in formaldehyde, and then stored in 75% ethanol.

The measurements and characters recorded from the individual were then compared with known specimens held in the Australian Museum Terrestrial Vertebrate Collection.

### Molecular methods

#### Samples, DNA extraction and sequencing

For the unknown *Pteropus* an ‘a’ and ‘b’ sample were taken and processed separately from other samples used in this study. Tissue samples were frozen at -20°C until DNA extraction. Genomic DNA was extracted using Qiagen DNeasy Blood and Tissue kit (Qiagen GmbH, Hilden, Germany) following standard protocols, or according to a high salt method [13].

To obtain a robust genetic reference dataset for the genus *Pteropus*, we obtained samples from 64 individuals, representing nine species from the Australian Museum Tissue Collection (Table 2). These specimens encompassed representatives from Australia (n = 28), the Pacific Islands (n = 35) and Indonesia (n = 1). Selected samples possessed information on sampling location, their identification had been confirmed by Australian Museum Terrestrial Vertebrate Collection staff and, where possible, vouchered specimens were available.

**Table 2:** Vouchered specimens and locations included in phylogenetic analyses for the identification of the unknown *Pteropus* sample. Specimens are deposited at the Australian Museum, Sydney, Australia. See S3 Table for details of GenBank sequences included in phylogenetic analyses.

Species	Label # in trees	Specimen number	Collection locality
*Pteropus alecto*	1	M.44046.001	Australia: Sydney, New South Wales
*Pteropus alecto*	2	M.44059.001	Australia: Sydney, New South Wales
*Pteropus alecto*	3	M.44312.001	Australia: Sydney, New South Wales
*Pteropus alecto*	4	M.44118.001	Australia: Sydney, New South Wales
*Pteropus alecto*	5	M.36131.001	Australia: Alstonville, New South Wales
*Pteropus alecto*	6	M.37534.001	Australia: Alstonville, New South Wales
*Pteropus alecto*	7	M.45032.001	Australia: Wollongbar, New South Wales
*Pteropus alecto*	8	M.32409.002	Australia: Lismore district, New South Wales
*Pteropus alecto*	9	M.36130.001	Australia: Wyrallah, New South Wales
*Pteropus alecto*	10	M.45036.001	Australia: Richmond Hill, New South Wales
*Pteropus alecto*	11	M.45028.001	Australia: Booyong, New South Wales
*Pteropus alecto*	12	M.44903.001	Australia: Clunes, New South Wales
*Pteropus alecto*	13	M.45035.001	Australia: Byron Bay, New South Wales
*Pteropus alecto*	14	M.37533.001	Australia: Rosebank, New South Wales
*Pteropus alecto*	15	M.37557.001	Australia: Lennox Head, New South Wales
*Pteropus alecto*	16	M.37565.001	Australia: Uki, New South Wales
*Pteropus alecto*	17	M.37564.001	Australia: Murwillumbah, New South Wales
*Pteropus alecto*	18	M.37563.001	Australia: Tweed district, New South Wales
*Pteropus alecto*	19	M.42865.001	Australia: Sydney, New South Wales
*Pteropus conspicillatus*	1	M.25292.002	Australia: Iron Range, Queensland
*Pteropus conspicillatus*	2	M.25291.002	Australia: Iron Range, Queensland
*Pteropus conspicillatus*	3	M.22564.004	Papua New Guinea: Hull Island
*Pteropus conspicillatus*	4	M.21584.003	Papua New Guinea: Hull Island
*Pteropus conspicillatus*	5	M.21582.004	Papua New Guinea: Hull Island
*Pteropus conspicillatus*	6	M.22539.004	Papua New Guinea: Hull Island
*Pteropus conspicillatus*	7	M.19446.001	Papua New Guinea: Muyua Island
*Pteropus conspicillatus*	8	M.21243.006	Papua New Guinea: Normanby Island
*Pteropus conspicillatus*	9	M.21669.001	Papua New Guinea: West Sepik Province
*Pteropus hypomelanus*	1	M.22543.005	Papua New Guinea: Hull Island
*Pteropus hypomelanus*	2	M.22541.005	Papua New Guinea: Hull Island
*Pteropus hypomelanus*	3	M.26845.005	Papua New Guinea: Fergusson Island
*Pteropus hypomelanus*	4	M.26846.001	Papua New Guinea: Fergusson Island
*Pteropus hypomelanus*	5	M.21571.008	Papua New Guinea: Sideia Island
*Pteropus hypomelanus*	6	M.21163.006	Papua New Guinea: Sideia Island
*Pteropus hypomelanus*	7	M.22536.006	Papua New Guinea: Itoh Island
*Pteropus hypomelanus*	8	M.19242.001	Papua New Guinea: Muyua Island
*Pteropus hypomelanus*	9	M.19989.001	Papua New Guinea: Muyua Island
*Pteropus neohibernicus*	1	M.21161.001	Papua New Guinea: New Britain
*Pteropus neohibernicus*	2	M.21240.001	Papua New Guinea: New Britain
*Pteropus ocularis*		M.29760.003	Indonesia: Seram
*Pteropus poliocephalus*	1	M.44021.001	Australia: Sydney, New South Wales
*Pteropus poliocephalus*	2	M.37027.002	Australia: Grafton, New South Wales
*Pteropus poliocephalus*	3	M.47114.001	Australia: Broadwater, New South Wales
*Pteropus poliocephalus*	4	M.47128.001	Australia: Lismore, New South Wales
*Pteropus samoensis*	1	M.23223.003	Fiji: Viti Levu Island
*Pteropus samoensis*	2	M.23224.003	Fiji: Viti Levu Island
*Pteropus samoensis*	3	M.23225.003	Fiji: Vanua Levu Island
*Pteropus samoensis*	4	M.23238.002	Fiji: Vanua Levu Island
*Pteropus samoensis*	5	M.24457.001	Fiji, Taveuni, Des Voeux Peak
*Pteropus samoensis*	6	M.23227.002	Fiji: Vanua Levu Island
*Pteropus samoensis*	7	M.23234.001	Fiji: Vanua Levu Island
*Pteropus samoensis*	8	M.24457.001	Fiji, Taveuni, Des Voeux Peak
*Pteropus scapulatus*	1	M.32440.003	Australia: Werrington Downs, New South Wales
*Pteropus scapulatus*	2	M.31806.001	Australia: Woy Woy, New South Wales
*Pteropus scapulatus*	3	M.37535.001	Australia: Ballina, New South Wales
*Pteropus tonganus*	1	M.22831.001	Fiji: Viti Levu Island
*Pteropus tonganus*	2	M.23218.003	Fiji: Vanua Levu Island
*Pteropus tonganus*	3	M.23220.001	Fiji: Vanua Levu Island
*Pteropus tonganus*	4	M.23222.001	Fiji: Vanua Levu Island
*Pteropus tonganus*	5	M.27155.001	Vanuatu: Efate Island
*Pteropus tonganus*	6	M.27144.003	Vanuatu: Mota Lava Island
*Pteropus tonganus*	7	M.27153.001	Vanuatu: Loh Island
*Pteropus tonganus*	8	M.23366.003	Solomon Islands: Nendo Island
*Pteropus tonganus*	11	M.23216.003	Fiji: Vanua Levu Island
Unknown *Pteropus*	a	M.47692.003	Australia: Hunters Hill, New South Wales
Unknown *Pteropus*	b	M.47692.004	Australia: Hunters Hill, New South Wales

Three mitochondrial (mtDNA) gene regions were amplified; *Control Region* (*CR*) [14], *Cytochrome b* (cytb) [15] and *Cytochrome C Oxidase subunit 1* (COI), modified from [16], and two nuclear genes; *Recombination Activating Gene 1* (RAG1) [17] and *von Willebrand Factor* (vWF)[18]. PCRs were carried out in 25 μl reactions using 100–500 ng of genomic DNA, 1 x Reaction Buffer (Bioline MyTaq Red Reagent Buffer; Bioline, Australia), 2 pmol of each primer and 0.5U Bioline MyTaq Red DNA polymerase. Negative controls were included in each PCR. Primer pairs and thermocycling conditions are shown in S1 Table. In the event that the first round of PCR failed to produce observable amplicons, a secondary reaction was performed using identical cycling conditions and undiluted PCR product from the primary reaction as template. PCR products were cleaned using ExoSap-IT (USB Corporation, Cleveland, Ohio, USA). Sequencing was resolved on an AB 3730xl Sequencer at the Australian Genome Research Facility Sydney.

Sequences were checked and edited with reference to chromatograms using Sequencher v 5.3.2 (Gene Codes Corporation, Ann Arbor, MI, USA) and novel sequences were lodged with GenBank under accession numbers MG924093—MG924334 (S2 Table). In addition, we obtained published sequence data for 43 *Pteropus* species (n = 179; S3 Table). All GenBank sequences were included, regardless of the availability of voucher material, although this was recorded (S3 Table). For species from Australia and the Oceania region we included all available sequences, and a maximum of 6 representative samples from species from elsewhere (following initial testing to ensure that these representative samples encompassed the available intraspecific diversity). Sequences were aligned using the Clustal X algorithm implemented in MEGA 7 [19].

#### Phylogenetic analyses

Phylogenetic relationships were estimated using Maximum Likelihood (ML) and Bayesian Inference (BI). The most appropriate model of evolution (GTR + I + G) was selected using the Akaike Information Criterion implemented in jMODELTEST [20]. ML trees were constructed using the .*pml* function in the package ‘phangorn’ [21] implemented in R (R Development Core Team, 2011). We used the GTR model with the proportion of invariant sites and rate variation optimised using the function *optim*.*pml*. Support for the branching topology was evaluated with 1000 bootstrap replicates. Bayesian inference analyses were implemented in MrBayes [22,23], with tree searches performed in two runs each of 10 million generations and four chains. For concatenated datasets, the parameters for BI were estimated separately from each partition of the data: mtDNA genes, 1^st^ and 2^nd^ codon positions; mtDNA genes, 3^rd^ codon position; nuclear genes, 1^st^ and 2^nd^ codon positions; and nuclear genes 3^rd^ codon position. Parameters and trees were sampled every 1000^th^ generation and convergence was assessed using TRACER [24]. Of the 10,000 sampled trees, the first 25% were discarded as burn-in. In each tree, sequences from related species were included as outgroups (see S3 Table).

Trees were obtained for each gene separately, and for combined mtDNA genes (*CR*, cytb, COI) and combined nuclear genes (vWF, RAG1). All *Pteropus* sequence data available from this study and those obtained from public databases were used for generating individual gene trees, while only sequence data from our samples and GenBank data from four mtDNA genomes were included in the combined trees (S2 and S3 Tables).

## Results

### Morphological data

The unknown flying-fox was a relatively small female, weighing 554 g. There was no lateral staining of the molar teeth enamel, minimal wear of the molar cusps, and no wear of canines, consistent with sub-adult bats between 1 and 2 years old (A. Divljan pers. comm.). Similarly, her nipples were furred and well regressed, indicative of a nulliparous animal [25]. Her forearm measurement (152.32 mm) was at the lower end of the scale reported for the three large Australian species: *P*. *alecto*, *P*. *poliocephalus*, and *P*. *conspicillatus* [25]; however, it is possible that the individual had not yet reached the full adult size. The individual was still larger in body size than would be expected for *P*. *scapulatus* of the same age. The left wing membrane was extensively damaged and torn, and there were skin scrapes on the right foot. The x-ray images generated prior to donation to the Australian Museum revealed no obvious bone fractures. Upon tissue sampling, the liver was found to be in a poor condition, possibly due to physical injury or trauma.

**Fig 1:**
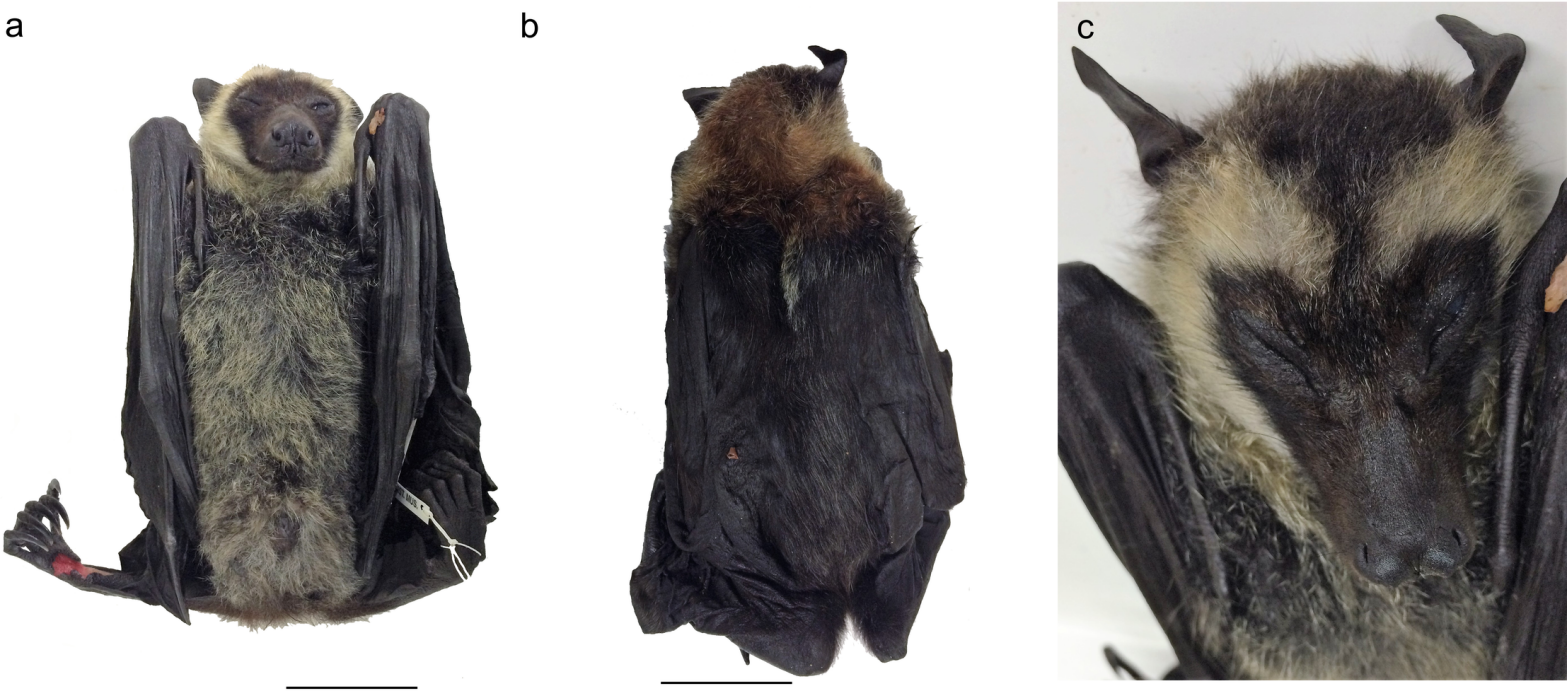
The unknown flying-fox (*Pteropus* sp.) found in Sydney in 2015. A. ventral; B. dorsal views and C. close-up of the face.

Morphologically this *Pteropus* possessed a unique combination of external features inconsistent with any of the Australian *Pteropus* species (Fig 1). The snout was long and slender with a dark (copper-red) tint, which is consistent with the shape and colouration found in most *P*. *alecto* [4]. Another feature reminiscent of *P*. *alecto* was the presence of hair on the ventral surface of the wing (Fig 1B). The individual possessed a pale straw-coloured collar/ring-like formation around the face, which has not previously been documented in any of the Australian flying-foxes. The body pelage was long and grizzled, similar to that of *P*. *poliocephalus*. However, unlike *P*. *poliocephalus*, the hair did not extend to the ankles (Fig 1A). Furthermore, the unusual overall appearance, and lack of rings around the eyes, reduced the possibility of the unknown *Pteropus* being *P*. *conspicillatus*. Similarly, the animal had no features resembling *P*. *scapulatus*, and the larger body size (for a sub-adult) further supported this. The combination of snout features and body pelage, however, suggested a possibility of the individual being a hybrid between *P*. *alecto* and *P*. *poliocephalus*. Our comparison of the morphology of study skins in the Australian Museum Terrestrial Vertebrate Collection, known published descriptions of the *Pteropus* species, and further consultation with researchers familiar with the genus also failed to identify this individual as any other known *Pteropus* species (e.g. Table 1).

### Molecular data and phylogeny

The concatenated mtDNA matrix was 1441 bp for nine species (S2 Table) and had 602 variable characters, 477 of which were parsimony informative. For the nuclear genes, vWF and RAG1, we obtained 1052 and 1085 bp of sequences, respectively, for nine species (S2 Table). Amplification success varied substantially between species and genes resulting in 60–66 sequences for mtDNA genes and 30 for nuclear genes (S2 Table).

Phylogenetic trees produced using BI and ML methods yielded similar branching structures and topologies for mtDNA (individual genes trees and concatenated trees) with moderate to strong support (BI: 0.70–1.00; ML: 70–100; Figs 2 and 3; S1 and S2 Figs). In general, the expected species groups were well supported with some species, such as *P*. *tonganus* and *P*. *samoensis*, exhibiting intraspecific structure. The relationships between some species, however, showed lower support and in some instances were inconsistent between trees (Fig 3 and S2 Fig). Both nuclear genes exhibited limited sequence divergence and low resolution in BI and ML trees, although it was possible to reliably separate several species, including distinguishing *P*. *alecto* from *P*. *poliocephalus* (Fig 4 and S3 Fig).

**Fig 2:**
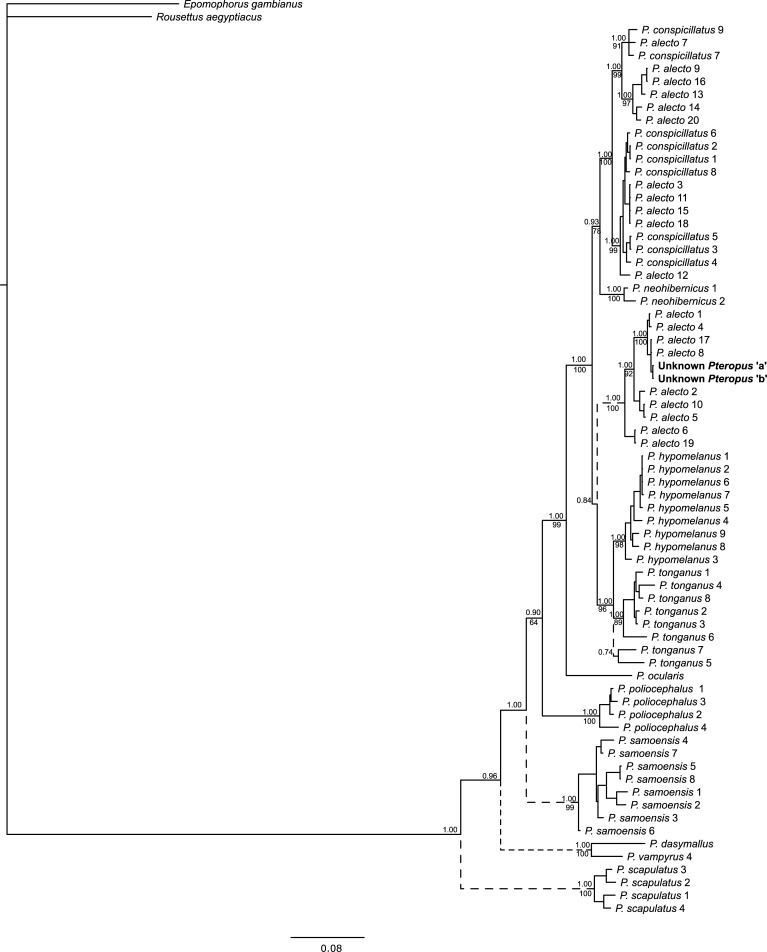
Bayesian tree based on concatenated mtDNA genes (*Cytochrome c oxidase 1*, *Control Region*, *Cytochrome b*). Support for clades are shown, with posterior probabilities shown above, and maximum likelihood values below branches. Branches with dashed lines were not recovered in the ML trees. See Table 2 and S2 and S3 Tables for locations.

**Fig 3:**
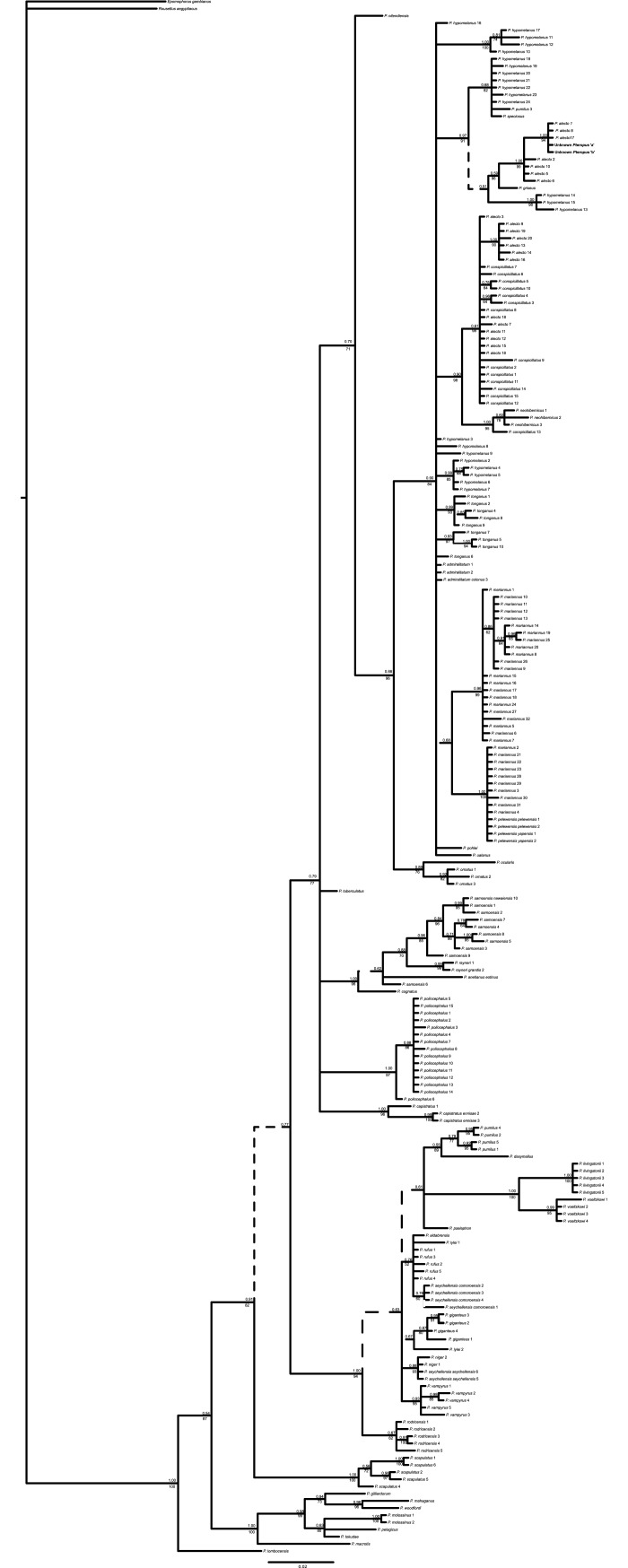
Bayesian tree based on 287 bp of *Cytochrome b*. Support for clades are shown, with posterior probabilities shown above, and maximum likelihood values below branches. Branches with dashed lines were not recovered in the ML trees. See Table 2 and S2 and S3 Tables for locations.

**Fig 4:**
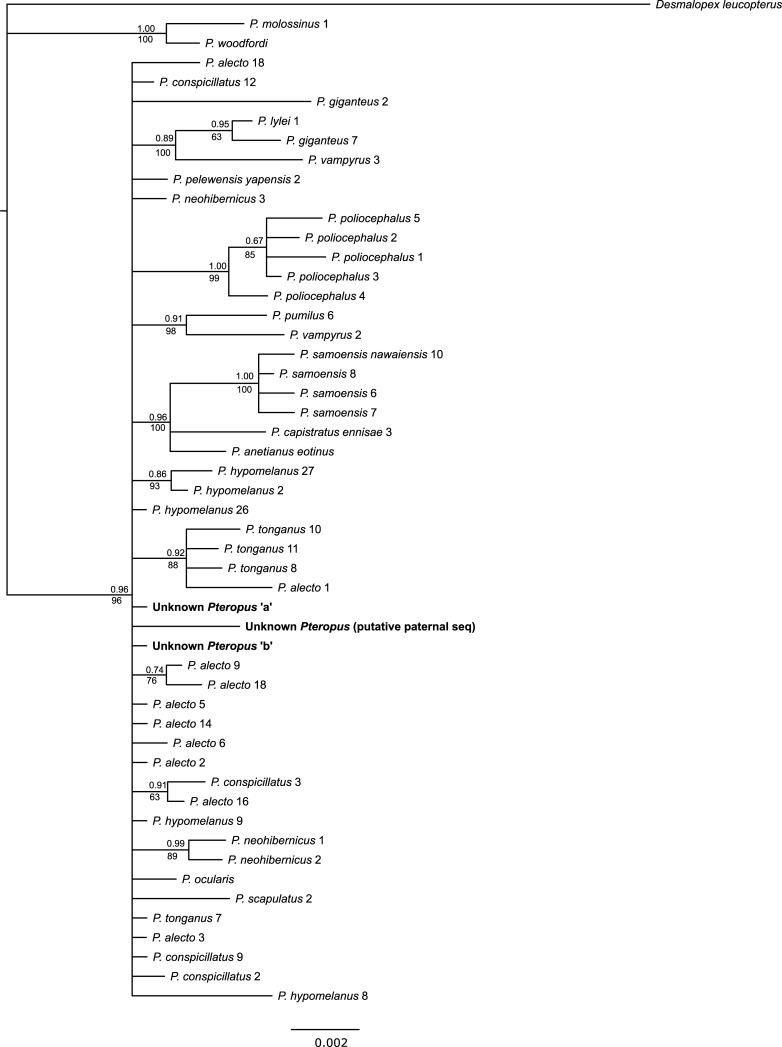
Bayesian tree based on 1052 bp of *von Willebrand Factor*. Support for clades are shown, with posterior probabilities shown above, and maximum likelihood values below branches. Branches with dashed lines were not recovered in the ML trees. See Table 2 and S2 and S3 Tables for locations.

Based on the concatenated mtDNA sequence (and each mtDNA gene individually) the mtDNA haplotype of the unknown *Pteropus* clustered within *P*. *alecto* haplotypes from Lismore and Sydney regions, NSW (Figs 2 and 3; S1 and S2 Figs). This clade was distinct from another lineage containing *P*. *alecto* sequences, which could not be distinguished from *P*. *conspicillatus*. Analysis using only sequences available from GenBank, for all gene regions but notably cytb, which contain the greatest representation of taxa indicated that the unknown *Pteropus* was divergent from the available sequences but most similar to *P*. *hypomelanus* and *P*. *griseus* (S4 Fig).

Analysis of RAG1 and vWF did not provide support for the unknown *Pteropus* representing a first generation hybrid between *P*. *alecto* and *P*. *poliocephalus*, as suggested by morphological comparisons. Even accounting for phasing in the nuclear genes, the unknown *Pteropus* did not possess nuclear sequence consistent with *P*. *poliocephalus* for either RAG1 (S3 Fig) or vWF (Fig 4). The possibility that the individual represents a second generation hybrid or backcross between *P*. *alecto* and *P*. *poliocephalus* could not be excluded based on these data. Similarly, a lack of resolution and differentiation between *P*. *alecto* and *P*. *conspicillatus* means we could not exclude the possibility of the unknown *Pteropus* representing a hybrid between these species, although it appears inconsistent with the results of the morphological assessment.

## Discussion

### Identification of the unknown *Pteropus*

Four potential alternatives for the origin of the unknown *Pteropus* were initially posited; (1) it was an undescribed species; (2) an unusual morph of a recognised Australian *Pteropus* species; (3) a hybrid; or (4) a vagrant from elsewhere in Australia or nearby Pacific Islands. The striking facial colouration is unlike any recognised species but, in general, the unknown *Pteropus* possessed morphological features typical of *P*. *alecto*, although its grizzled body pelage is more characteristic of *P*. *poliocephalus*. The strong resemblance to *P*. *alecto* is consistent with the results of mtDNA analyses which indicated that the maternal lineage of the unknown *Pteropus* was *P*. *alecto*.

The combination of morphological characteristics typically associated with *P*. *alecto* and *P*. *poliocephalus* were suggestive of hybridisation. However, the unknown *Pteropus* did not possess furred ankles, contrary to the few previously identified *P*. *alecto* / *P*. *poliocephalus* hybrids [6]. Even though resolution in the nuclear genes was poor, it was sufficient to distinguish *P*. *poliocephalus* from other species, including *P*. *alecto*. Thus, based on our results the unknown *Pteropus* does not appear to be a first generation hybrid between *P*. *alecto* and *P*. *poliocephalus*, but it was not possible to exclude second generation hybrids or backcrosses using these data. Fertile hybrids between these two species have been reported and a later generation hybrid may explain the combination of morphological traits which are inconsistent with previously reported F1 hybrids [6]. Hybridisation is also suspected to occur between *P*. *alecto* and *P*. *conspicillatus*, but like previous studies we found high levels of genetic similarity between the two species, which hampers investigation of hybridisation [3,6]. Thus, we could not exclude this possibility based on genetic data. However, a hybrid of *P*. *alecto* and *P*. *conspicillatus* would not explain the grizzled pelage of the unknown *Pteropus*. More thorough investigation using multiple nuclear markers, and determination of allele frequencies in both allopatric and sympatric populations will be required to conclusively determine the potential hybrid status of this specimen.

The mtDNA haplotype of the unknown *Pteropus* clustered with *P*. *alecto* from the Lismore and Sydney region. There was no indication of intraspecific phylogeographic structure within *P*. *alecto*, although two clades were evident, most likely a result of hybridisation with *P*. *conspicillatus*. The distribution of *P*. *alecto* has been expanding south and has been recorded in Sydney since 2007 [26], with individuals establishing permanently in Sydney since 2010 (A. Divljan, pers. comm.). Thus, this individual may have originated in the Sydney region or from further north.

Based on our genetic and morphological analyses we suggest the unknown *Pteropus* specimen is most likely either an unusual morph of *P*. *alecto* or potentially the result of introgression between *P*. *alecto* and *P*. *poliocephalus*. Introgression from another *Pteropus* species, however, could not be ruled out.

### Reference data and species identification

Publicly available databases, such as GenBank and BOLD, provide a large and rapidly expanding set of nucleotide sequence data for a wide array of taxonomic groups, which can be used as reference data. However, there is the potential for misidentifications, mislabelling, contamination and sequencing errors in these data that can confound species identification [27]. GenBank provides a vast amount of potential reference data, but there is limited quality control and erroneous sequences are present [28,29]. Furthermore, many sequences have been obtained from non-vouchered specimens, which often cannot be verified [30]. Several *Pteropus* sequences obtained from GenBank have been identified as potentially incorrect both in this and previous studies [31] (see also *Pteropus* phylogeny below). In contrast, BOLD provides a highly curated database, which only contains vouchered specimens, providing more confidence to species identification. However, the range of data available is limited and while BOLD accepts data from a range of gene regions (~150, http://www.boldsystems.org/), it focuses on a single animal barcoding gene (COI) and hence there may be insufficient data for species identification in complex or hybridising groups, such as *Pteropus*.

Limited representation of taxa for comparison in species identification can present a significant impediment to robust species identification, particularly in the presence of erroneous reference species, in complex taxonomic groups (incl. hybridisation and recent radiations) and widespread taxa, where there is the potential for significant biogeographic structuring. Despite the availability of over 280 sequences from 43 *Pteropus* species in publicly available databases for one gene in this study (cytb), which included three *P*. *alecto* sequences from a range of locations, a divergent *P*. *alecto* lineage was not represented. For the unknown *Pteropus* investigated here, the absence of this lineage meant that, based solely on publicly available data, the individual appeared distinct from all currently available *Pteropus* species and most similar to *P*. *griseus* from Indonesia or *P*. *hypomelanus*, an insular species distributed from India to Melanesia (S4 Fig). The available data from other genes provided no evidence to suggest the existence of two *P*. *alecto* lineages (from either mtDNA or nuclear genes). Thus, very different conclusions may have been drawn based only on publicly available data, with the unknown *Pteropus* potentially representing a previously unknown species, or a vagrant *P*. *griseus* or *P*. *hypomelanus*, blown in during extreme storm activity.

Our results highlight the importance of museum collections and developing large and robust reference datasets for species identification. The complex relationship, and potential introgression of mtDNA between *P*. *alecto* and *P*. *conspicillatus* was only apparent following the addition of 28 *P*. *alecto* and *P*. *conspicillatus* vouchered reference samples. Ideally, it would be possible to recommend a standardised number of samples required for robust species identifications. However, determining a minimum number of samples sufficient to provide robust species identification is not straightforward and will typically be species-specific, depending on the complex demographic and biological processes such as life-history traits and dispersal capabilities, and the potential for hybridisation, which may not be well understood. Rather than recommend a standard number of samples, we suggest that it is more important that reporting is standardized, and that in providing species identification researchers are aware of, account for and report the potential limitations of the available data and the potential impacts on the robustness and accuracy of species identification. In the long term, the availability of extended barcodes and genome-wide data will provide a stronger framework for detecting and accounting for complex biological processes, such as hybridisation and introgression (e.g. [32]).

### Species identification and management in *Pteropus*

The conservation and management of *Pteropus*, like many bat species, is complex and often contentious. DNA-based identification from faecal material can provide an assessment of the species occurring at particular roosting sites, with minimal disturbance to the animals [33,34]. The ability to accurately monitor species can also aid in disease control as several *Pteropus* species have been identified as reservoir hosts for numerous recently emergent pathogens, such as Hendra virus (henipaviruses) and Australian bat lyssavirus (lyssaviruses) [35–37]. The daily large-scale movement of individuals from roosting sites to feeding grounds can lead to collisions with wind turbines [38] and with departing and landing aircraft [39,40], presenting a significant safety risk even to large aircraft, particularly in Australia [41], and identification of the species involved is often not possible without the application of DNA-based identification. Accurate species identification and delineation of *Pteropus* species for conservation is also critical at international scales (e.g. International Convention on International Trade in Endangered Species of Flora and Fauna (CITES), International Union for Conservation of Nature (IUCN) Red Lists and quarantine classifications). For example, all *Pteropus* species are listed on CITES but several species, especially in southeast Asia are found in the illegal wildlife trade [30,42]. The importance of conservation and the vulnerability of *Pteropus* species to global extinction is further highlighted by the historic extinction of a number of Australian and Southwest Pacific *Pteropus* (e.g. *P*. *allenorum*, *P*. *brunneus*, *P*. *coxi*, and *P*. *pilosus*) [43].

### *Pteropus* phylogeny

The relationships between *Pteropus* species are complex and several remain poorly resolved [31]. The addition of sequences from 64 new vouchered specimens by this study to increase the available reference data also provides new insights into the phylogenetic relationships.

*Pteropus alecto* and *P*. *conspicillatus* have previously been noted to be paraphyletic. This may be the results of incomplete lineage sorting and/or introgression between closely related species [3,31]. Our results revealed a previously unreported lineage of *P*. *alecto* mtDNA haplotypes distinct from all other *Pteropus* species and previously described *P*. *alecto* haplotypes, which form a mixed clade with *P*. *conspicillatus*. This suggests the long-term introgression of *P*. *alecto* mtDNA haplotypes into *P*. *conspicillatus* populations, rather than incomplete lineage sorting. The geographical spread of *P*. *alecto* with *P*. *conspicillatus*-like haplotypes suggests that introgression is not localised but may occur wherever these species occur in sympatry. However, based on the results of this study (and [3]), hybridisation appears unidirectional, with the introgression of *P*. *conspicillatus* mtDNA into *P*. *alecto* populations. This is consistent with the presence of some barriers to hybridisation, which may include segregation resulting from differences in selection of roosting sites [3] and pre-zygotic mating avoidance mechanisms such as species-specific secretions [44]. Given the presence of potentially widespread introgression, it is possible that some morphological variation previously noted in *P*. *alecto*, notably individuals possessing light colouration around the eyes [6], may be due to misidentification of hybrid individuals. To date, markers examined have displayed insufficient resolution to satisfactorily assess hybridisation and introgression (this study and [6]). Studies investigating large numbers of nuclear loci (e.g. microsatellites or SNPs) across the distribution of both species, including sympatric and allopatric populations will be required to determine the extent of hybridisation between these two species (e.g. [45–47]).

The placement of *P*. *ocularis*, which has only been recorded on three islands in Indonesia, has remained uncertain due to limited genetic data (only 12S are available; [31]). Based on our data *P*. *ocularis* does appear distinct from other *Pteropus* species and, as suggested by Almeida *et al*. [31], likely represents its own species group. However, based on the comparisons of cytb sequences here, *P*. *ocularis* appears most similar to *P*. *ornatus* (which also forms its own species group) rather than *P*. *lombocensis* as previously suggested [31].

*Pteropus* are highly mobile species, capable of traversing large distances and hence may not be expected to exhibit substantial intraspecific genetic structure. In the Australian samples there was no indication of intraspecific genetic structuring (Figs 2 and 3) [3]. This is not surprising given several studies have demonstrated their ability to move large distances [3,48,49]. For instance, movements totalling 2000 km over 12 months have been recorded in both *P*. *poliocephalus* and *P*. *conspicillatus*. In contrast, we noted intraspecific structuring in some of the insular species, notably *P*. *tonganus* and *P*. *samoensis*, which may be associated with their movement ability. The wing morphology of *P*. *tonganus* is associated with long, fast flights [50]. Movements within islands up to 22 km have been recorded [51] and water does not appear to present a substantial barrier within archipelagos [52]. However, our results indicate some intraspecific structure, with samples from Fiji, the Solomon Islands and Vanuatu appearing to form separate clusters, suggesting longer distance movements between archipelagos may be infrequent. Unlike *P*. *tonganus*, *P*. *samoensis* wing morphology indicates soaring flight [50], but to the best of our knowledge no detailed studies on flight distances exist. Our results suggest much stronger intraspecific structure within *P*. *samoensis*, with clusters apparent within the Fijian Islands, representing Vanua Levu, Viti Levu and Taveuni Islands. This may indicate that water presents a greater barrier to movement for the soaring flight of *P*. *samoensis*. Further research into the intraspecific variation in these species, and the differences between them may provide insights into to the biogeography of the region and the dispersal differences between these two ecologically similar species.

*Pteropus hypomelanus* is widespread from India in the west of its range to Melanesia in the east. Genetically, *P*. *hypomelanus* appears polyphyletic and the relationships between populations throughout these locations and with other *Pteropus* species appear complex [31]. There was no evidence of substructure within the PNG samples included in this study. However, these samples formed a distinct cluster separate from previously published *P*. *hypomelanus* samples from elsewhere in the range. Thus, there appear to be at least four distinct genetic groupings of *P*. *hypomelanus*: PNG, Malaysia, Philippines, and a fourth group, which consists of bats of unknown origin housed at the Lubee Conservation Centre (USA), although at least one may be from Pulau Panjang from the northwest coast of Java [31]. Given the levels of genetic differentiation and location of these clades throughout the phylogenetic tree (Fig 3) it is probable that *P*. *hypomelanus* represents multiple species rather than a single widespread, variable species. Hybridisation and the introgression of mtDNA haplotypes is also possibly contributing to these patterns. Regardless, additional research, using both mtDNA and nuclear markers is required to elucidate the relationships not only amongst populations of *P*. *hypomelanus*, but also with closely related species.

## Conclusions

Using a combination of morphological- and molecular- (both mtDNA and nuclear DNA) based identification we propose that the most likely origin of a morphologically unique *Pteropus* found in Sydney following large and widespread storm activity, is an unusual *P*. *alecto* originating from NSW and potentially resulting from introgression from another *Pteropus* species. Surprisingly, given the relatively large amount of sequence data already available for *Pteropus*, we found evidence for a novel *P*. *alecto* mtDNA lineage that was distinct from *P*. *alecto* mtDNA haplotypes that were publicly available. The latter could not be separated from *P*. *conspicillatus* and our data provide further evidence that there is widespread introgression of mtDNA from *P*. *conspicillatus* into *P*. *alecto* populations. Our data highlight the importance of utilising extensive reference data, including multiple vouchered specimens for each species, to encompass both intraspecific and interspecific variation sufficient to provide accurate and robust species identification. Furthermore, the additional museum vouchered reference specimens and data added here also provide insights into some of the complex relationship between *Pteropus* species and suggests intraspecific biogeographical structure occurs in several insular species which requires further investigation.

## Supporting information

S1 Table Genes, primers and PCR cycling conditions used in this study. (DOCX)

S2 Table Samples and gene regions sequenced (with GenBank accession numbers) from the Australian Museum, Sydney, Australia. (XLSX)

S3 Table Sequences obtained from GenBank and used in analysis presented in this study. (XLSX)

S1 Fig Bayesian tree based on 657 bp of *cytochrome c oxidase subunit 1*. Support for clades are shown, with posterior probabilities shown above, and maximum likelihood values below branches. Branches with dashed lines were not recovered in the ML trees. See Table 2 and S2 and S3 Tables for locations.
(EPS)

S2 Fig Bayesian tree based on 497 bp of mitochondrial DNA *control region*. Support for clades are shown, with posterior probabilities shown above, and maximum likelihood values below branches. Branches with dashed lines were not recovered in the ML trees. See Table 2 and S2 and S3 Tables for locations.
(EPS)

S3 Fig Bayesian tree based on 1085 bp of *Recombination activating gene 1* (RAG1). Support for clades are shown, with posterior probabilities shown above, and maximum likelihood values below branches. Branches with dashed lines were not recovered in the ML trees. See Table 2 and S2 and S3 Tables for locations.
(EPS)

S4 Fig Bayesian tree showing the relationship between the unknown *Pteropus* sample and sequences obtained from GenBank for 287 bp of cytochrome b. Support for clades are shown, with posterior probabilities shown above, and maximum likelihood values below branches. Branches with dashed lines were not recovered in the ML trees. See S3 Table for locations.
(TIF)
