# Supplementary material for: Unmasking the complexity of species identification in Australasian flying-foxes
Source: PLoS One. 2018 Apr 10;13(4):e0194908. doi: 10.1371/journal.pone.0194908 (PMC5892893; doi:10.1371/journal.pone.0194908)
Supplement: S1 Table — (DOCX) [file pone.0194908.s001.docx]

| **Target Gene** | **Primer Sequence (5’ → 3’)** | **Length (bp)** | **Initial Denaturation**  **( ^o^C)** | **Denaturation**  **( ^o^C)** | **Annealing**  **( ^o^C)** | **Extension**  **( ^o^C)** | **No. of Cycles**  **( ^o^C)** | **Final Extension**  **( ^o^C)** | **References** |
| --- | --- | --- | --- | --- | --- | --- | --- | --- | --- |
| Cytochrome b | AAAAAGCTTCCATCCAACATCTCAGCATGATGAAA  AAACTGCAGCCCCTCAGAATGATATTTGTCCTCA | ~307 | 94  (3 min) | 94  (20 sec) | 55  (40 sec) | 72  (40 sec) | 38 | 72  (5 min) | L14841 & H15149 in Kocher et al 1989 Proc Natl Acad Sci |
| Cytochrome c oxidase subunit 1 | CTCAACCAACCACAAAGACATCGG  TAGACTTCTGGGTGGCCGAAGAATCA | ~660 | 94  (3 min) | 94  (20 sec) | 55  (40 sec) | 72  (40 sec) | 38 | 72  (5 min) | BAK1490 & BAK2198 in this study (modified from Folmer et al 1994 Mol Mar Biol Biotechnol) |
| mtDNA control region | GCTGAGGTTCTACTTAAACT  GAGATGTCTTATTTAAGGGG | ~470 | 94  (5 min) | 94  (30 sec) | * 65-55  (30 Sec) | 72  (45 sec) | 30 | 72  (7 min) | RodmtU & RodmtL in Brown et al 2011 Conserv Genet |
| Recombination-activating gene 1 | GCTTTGATGGACATGGAAGAAGACAT GAGCCATCCCTCTCAATAATTTCAGG | ~1120 | 94  (5 min) | 94  (30 sec) | * 65-55  (30 Sec) | 72  (45 sec) | 30 | 72  (7 min) | RAG1F1705 & RAG1R2864 in Teeling et al 2000 Nature |
| Von Willebrand factor | CTGTGATGGTGTCAACCTCACCTGTGAAGCCTG  TCGGGGGAGCGTCTCAAAGTCCTGGATGA | ~1200 | 94  (5 min) | 94  (30 sec) | * 65-55  (30 Sec) | 72  (45 sec) | 30 | 72  (7 min) | vWF-AF & vWF-BR2 in Porter et al 1996 Mol Phyl Evol |

**S1 Table. Genes, primers and PCR cycling conditions used in this study**

* A touchdown step was performed with an annealing temperature decrement of 2 ^o^C per cycle for 5 cycles**.** Cycling was then performed as indicated at the final annealing temperature reached (55^o^C). No other conditions were modified.
