# Supplementary figures and images for: Unmasking the complexity of species identification in Australasian flying-foxes
Source: PLoS One. 2018 Apr 10;13(4):e0194908. doi: 10.1371/journal.pone.0194908 (PMC5892893; doi:10.1371/journal.pone.0194908)

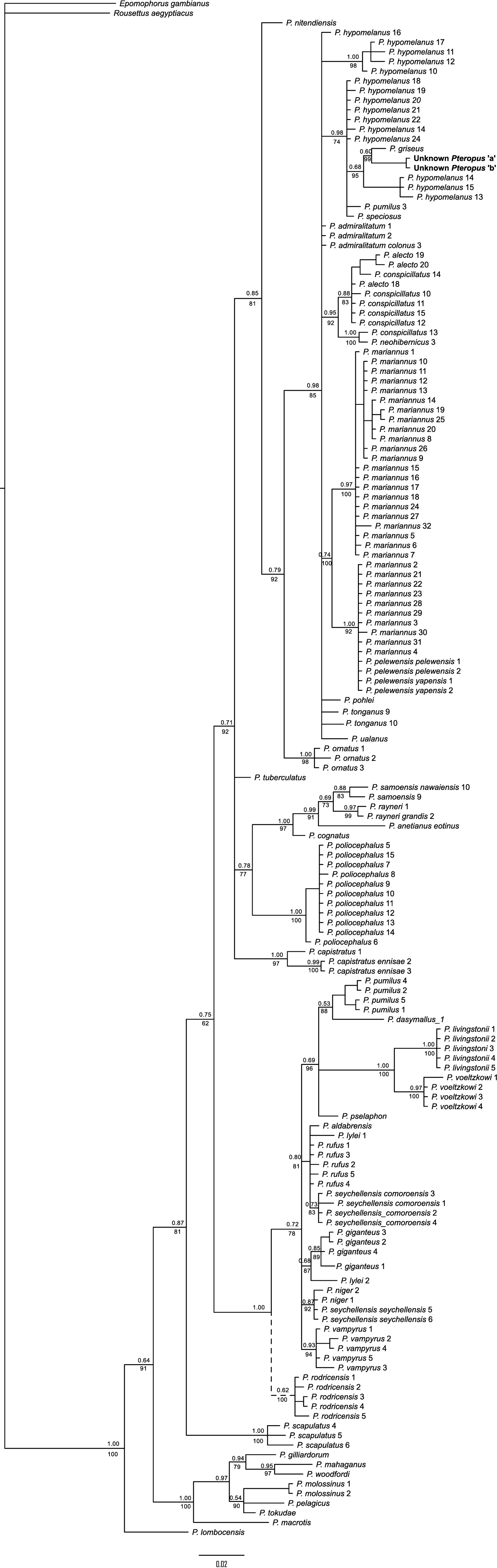

Supplement: S4 Fig — Support for clades are shown, with posterior probabilities shown above, and maximum likelihood values below branches. Branches with dashed lines were not recovered in the ML trees. See S3 Table for locations. (TIF) [file pone.0194908.s007.tif]
